# Insights on Alterations to the Rumen Ecosystem by Nitrate and Nitrocompounds

**DOI:** 10.3389/fmicb.2016.00228

**Published:** 2016-03-04

**Authors:** Elizabeth A. Latham, Robin C. Anderson, William E. Pinchak, David J. Nisbet

**Affiliations:** ^1^Department of Animal Science, Texas A&M UniversityCollege Station, TX, USA; ^2^Texas A&M AgriLife ResearchVernon, TX, USA; ^3^Food and Feed Safety Research Unit, Southern Plains Agricultural Research Center, United States Department of Agriculture, Agricultural Research ServiceCollege Station, TX, USA

**Keywords:** nitrate, nitrocompounds, rumen, methane reduction, nitrate toxicity

## Abstract

Nitrate and certain short chain nitrocompounds and nitro-oxy compounds are being investigated as dietary supplements to reduce economic and environmental costs associated with ruminal methane emissions. Thermodynamically, nitrate is a preferred electron acceptor in the rumen that consumes electrons at the expense of methanogenesis during dissimilatory reduction to an intermediate, nitrite, which is primarily reduced to ammonia although small quantities of nitrous oxide may also be produced. Short chain nitrocompounds act as direct inhibitors of methanogenic bacteria although certain of these compounds may also consume electrons at the expense of methanogenesis and are effective inhibitors of important foodborne pathogens. Microbial and nutritional consequences of incorporating nitrate into ruminant diets typically results in increased acetate production. Unlike most other methane-inhibiting supplements, nitrate decreases or has no effect on propionate production. The type of nitrate salt added influences rates of nitrate reduction, rates of nitrite accumulation and efficacy of methane reduction, with sodium and potassium salts being more potent than calcium nitrate salts. Digestive consequences of adding nitrocompounds to ruminant diets are more variable and may in some cases increase propionate production. Concerns about the toxicity of nitrate's intermediate product, nitrite, to ruminants necessitate management, as animal poisoning may occur via methemoglobinemia. Certain of the naturally occurring nitrocompounds, such as 3-nitro-1-propionate or 3-nitro-1-propanol also cause poisoning but via inhibition of succinate dehydrogenase. Typical risk management procedures to avoid nitrite toxicity involve gradually adapting the animals to higher concentrations of nitrate and nitrite, which could possibly be used with the nitrocompounds as well. A number of organisms responsible for nitrate metabolism in the rumen have been characterized. To date a single rumen bacterium is identified as contributing appreciably to nitrocompound metabolism. Appropriate doses of the nitrocompounds and nitrate, singly or in combination with probiotic bacteria selected for nitrite and nitrocompound detoxification activity promise to alleviate risks of toxicity. Further studies are needed to more clearly define benefits and risk of these technologies to make them saleable for livestock producers.

## Introduction

Nitrate and other oxidized nitrocompounds are scientifically pursued due to their toxicity and ability to reduce enteric methane emissions by ruminants. Methane production by microbes within the rumen is recognized as a fermentative inefficiency resulting in the loss of 2–12% of the gross energy consumed by the host (Johnson and Johnson, 1995). Environmentally, methaneis a significant greenhouse gas and strategies are sought to reduce its emission from livestock, which in the United States accounts for 95% of anthropogenic methane emissions arising from enteric fermentation (US EPA, 2012). However, despite its negative association with energy retention and greenhouse gas emissions, methanogenesis plays an important ecological role in the rumen. Archaea consume hydrogen emitted by bacterial and protozoal hydrogenases functioning to reoxidize reduced nucleotides produced during glycolysis and other catabolic pathways (Ellis et al., 1990; Miller, 1995). Methanogenesis functions to maintain a low partial pressure of hydrogen, which at partial pressures above 1 kPa promote end-product inhibition of NADH oxidoreductase thereby disrupting the oxidation of NADH and depleting concentrations of NAD to levels that inhibit fermentation (Miller, 1995; Van Nevel and Demeyer, 1996; Hegarty and Gerdes, 1999).

It is generally recognized that the most effective methane-inhibiting interventions provide alternative mechanisms for maintaining low partial pressures of dihydrogen within the rumen. A variety of electron accepting substrates are available for use as alternative electron acceptors for anaerobic respiration in the rumen, including unsaturated fatty acids, nitrate, sulfate or fumarate (Leng, 2014). From a thermodynamic perspective, however, the use of nitrate is particularly attractive because the dissimilatory reduction to ammonia is energetically more favorable (ΔG^0^′ = −600 kJ/mol) than the reduction of carbon dioxide to methane (ΔG^0^′ = −136 kJ/mol) and the reduction of other electron acceptors available in an anaerobic environment (Thauer et al., 1977; Table 1). Moreover, supplemental nitrate could under certain dietary conditions serve and perhaps even replace nonprotein nitrogen sources such as urea to support microbial protein synthesis in the rumen (Carver and Pfander, 1974; Sophea and Preston, 2011; Li et al., 2012; Silivong et al., 2012; Thanh et al., 2012). Theoretically, the consumption of four electrons with the reduction of nitrate to nitrous oxide, and potential consumption of an additional electron for the reduction of nitrous oxide to dinitrogen (N_2_) via denitrification, could also serve as a metabolic route for electron disposal to the reduction of nitrate. However, from an energetic perspective, the reduction of nitrite to ammonia is slightly more favorable thermodynamically than the reduction of nitrite to nitrous oxide (436 kJ/mol vs. 453 kJ/mol), and thus ammonia is the prevalent reduction product in the rumen (Table 1).

**Table 1:** **Standard molar Gibbs Free energy for reductive processes**.

**Reaction^a^**	**Electrons**	***E*° (mV)**	**−ΔG° (kJ/mol)**
Carbon dioxide reduction to methane	−8	−244	131
Fumarate to succinate	−2	33	86
Oxygen to water	−2	818	228
Nitrate reduction to ammonia	−8	–	599.6
Nitrate reduction to nitrite	−2	433	163.2
Nitrite reduction to ammonia	−6	363	436.4
Nitrate reduction to nitrogen gas	−5	–	1120
Nitrate reduction to nitric oxide	−3	350	147
Nitric oxide reduction to nitrous oxide	−1	1175	306.1
Nitrous oxide reduction to nitrogen gas	−1	1355	341.4

a *Adapted from Thauer et al. (1977)*.

Early work by Anderson et al. (1993, 1997) revealed that like nitrate and nitrite, the naturally-occurring nitrocompounds 3-nitro-1-propionate and 3-nitro-1-propanol and industrially-produced nitroethane may also serve as electron acceptors within rumen microbial populations. However, in addition to serving as alternative electron acceptors these nitrocompounds also exert a direct inhibition of ruminal methanogenesis (Anderson and Rasmussen, 1998; Gutierrez-Bañuelos et al., 2008). Consumption of electrons, at least with the reduction of naturally occurring nitrocompounds and nitroethane, occurs more slowly than the direct inhibition mechanism and requires *in situ* enrichment of competent nitro-reducing bacteria that are normally present at low numbers. The biological processes involved in the direct chemical inhibition of methane production by the short chain nitrocompounds are ill-defined. It has been speculated that this could occur via inhibition of electron transfer reactions like the nitroethanol-caused inhibition of electron transfer between ferredoxin and hydrogenase (Angermaier and Simon, 1983; Anderson et al., 2008). A number of other short chain nitrocompounds have been tested *in vitro* and while most if not all have been found to effectively inhibit ruminal methane production at present only a few have been found to be suitable electron acceptors for supporting growth of nitro-reducing bacteria. Alternatively, inhibition of methyl-coenzyme M reductase of methanogenic bacteria has been postulated for the recently identified inhibitor, 3-nitrooxypropanol, as well as some other nitro-oxy-compounds (Martínez-Fernández et al., 2014; Prakash, 2014). Thus, these nitro-oxy compounds, which possess an oxygen atom binding the nitro-group at the number 3 carbon, not only differ structurally from the short chain nitrocompounds discussed above, but probably in their mode of action as well.

Multiple literature reviews on the toxicological aspects and methane reducing potential of feeding nitrate to ruminants have been published recently including excellent works by Lee and Beauchemin (2014) and Leng (2008). Consequently, the present work focuses our discusssions on the microbiological response to nitrate and nitrocompound supplementation.

## Nitrate and nitrite metabolism within the rumen

Microbial reduction of nitrate can occur by dissimilatory and assimilatory processes. The genes involved, their regulation and the energetics of these pathways substantialy differ (Table 2). The assimilatory nitrate reduction pathway consumes energy to reduce nitrate to ammonia as a nitrogen source for microbial protein synthesis and is repressed by ammonia (Moreno-Vivián et al., 1999). Consequently, the functional role of this process is largely unnecessary in environments like the rumen where the availability of ammonia may down regulate this activity. Dissimilatory nitrate reduction, on the other hand, is an energy generating process that is distributed widely amoung obligate and facultative anaerobic bacteria (Thauer et al., 1977). Within the rumen, dissimilatory nitrate reduction occurs primarily via a two-step pathway where nitrate is first reduced to nitrite, which can accumulate as an intermedite before it is ultimately reduced to ammonia. Enzymes involved in dissimilatory nitrate reduction include membrane bound and periplasmc nitrate reductases encoded by *nar and nap* genes and nitrite reductases encoded by *nir* and *nrf* genes (Thauer et al., 1977; Moreno-Vivián et al., 1999; Table 2). Dissimilatory nitritereduction occurs on the outer cytoplasmic membrane and depending on the organism, consumes electrons via the oxidation of reduced electron carriers such as NADH or FADH and upon subsequent transfer of these electrons to the respiratory electron transport system they can be used to reduce and thus detoxify nitrite (Thauer et al., 1977; Moreno-Vivián et al., 1999). Certain bacteria may lack a complete functional electron transport chain yet be able to reduce nitrate to nitrite and sometimes to ammonia via reductive reactions with the incomplete chain or other electron carriers without generating ATP. In such cases, these bacteria are thought to still gain an energetic benefit via more effective disposal of electrons and therefore more efficient recycling of NAD via oxidation of NADH produced during glycolysis (Hasan and Hall, 1975).

**Table 2:** **Microbial nitrogen metabolism in the rumen^a^**.

**Oxidation state**	+**5**		**+3**	**+2**	**+1**	**−3**
Dissimilatory nitrate reduction	NO_3_	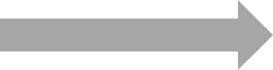	NO_2_	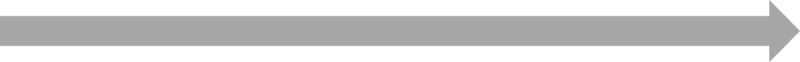		NH_4_
		*nar/nap*			*nir/nrf*	
Assimilatory nitrate reduction	NO_3_	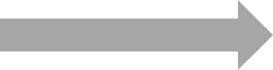	NO_2_	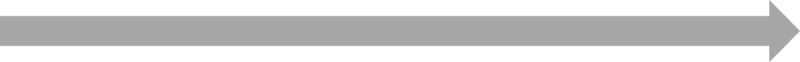		NH_4_
		*nar/nas*			*nir*	
Denitrification	NO_3_	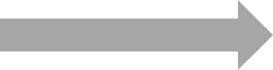	NO_2_	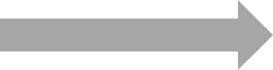 NO	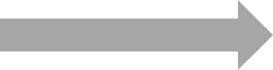 N_2_O	
		*nar/nap*		*Nir*	*nor*	

a *NO_3_, nitrate; NO_2_, nitrite; NO, nitric oxide; N_2_O, nitrous oxide; NH_4_, ammonium*.

Denitrification is another pathway for dissimilatory nitrate reduction, yet despite evidence for the presence of the denitrifying genes (*nir, nor*, and *nos*) within the rumen eubacterial and archaeal metagenome (Zumft and Kroneck, 2007; Brulc et al., 2009), this process is not considered to contribute appreciably to ruminal nitrate reduction (Jones, 1972; Kaspar and Tiedje, 1981; Leng, 2008; Table 2). In a metagenomic study by Brulc et al. (2009), 85 occurances of genes associated with denifrication and nitrogen fixation were tagged in bovine rumen metagenomics samples compared to the occurance of 636 genes contributing to nitrate and nitrite ammonification and 1233 total genes contributing to nitrogen metabolism. Thus, the authors concluded that denitrification and nitrogen fixation activities were likely inconsequential in the rumen. However, genetically this accounts for 7% of the genes involved in nitrogen metabolism and while clearly not dominant, their contribution cannot be completely ruled out.

Few studies have measured ruminal nitrous oxide accumulation in response to nitrate supplementation, however, in studies that have the amounts produced were found to vary considerably. Nitrous oxide concentrations equivalent to 0.3% the amount of added nitrate or nitrite were measured within nitrate- or nitrite-supplemented *in vitro* rumen fluid incubations and *in vivo* from the rumen of nitrate-supplemented [as 5 Ca(NO_3_)${}_{2}^{.}$NH_4_NO${}_{3}^{.}$10 H_2_O] sheep (Kaspar and Tiedje, 1981; de Raphélis-Soissan et al., 2014). However, Petersen et al. (2015) found nitrous oxide emissions account for as much as 3.4% of added nitrate (21 g NO_3_kg^−1^ dry matter fed to adapted dairy cows, nitrate type unknown). The later evidence suggests that denitrification may be contributing to nitrous oxide production in the rumen rather than being produced simply as a nonspecific byproduct of dissimilatory nitrite reduction by the nir nitrite reductase as postulated by Kaspar and Tiedje (1981). Potential bacterial denitrifiers are *Pseudomonas aeruginosa* and certain species of *Propionibacterium* and *Nitrosomonas*, which in the absence of added nitrate may be considered transient or minor colonizers of the rumen (Bryant, 1959; Duncan et al., 1999; Mitsumori et al., 2002; Arai et al., 2003). Future meta-transcriptomic analysis and/or RT-qPCR combined with nitrous oxide emission measurements could determine the relative abundance and gene expression of the denitrification pathways within the rumen of animals adapted to different types and amounts of nitrate.

## Microbial response to nitrate

Microbial nitrate and nitrite metabolism in the rumen is a paradox in that it enables detoxification, but also results in the formation of toxicants (Table 3). Since these microbes are symbionts to the ruminant host, an understanding of both their response in terms of gene expression and community structure and their susceptibility to added nitrate and nitrite becomes exceedingly important. Exposure of unadapted microbial populations to high intakes of dietary nitrate results in the rapid induction of nitrate reducing activity, as evidenced by >14-fold increases in activity within 4 h of first exposure, as well as the gradual selection of highly competent nitrate and nitrite reducing rumen bacteria (Allison and Reddy, 1984). Induction of nitrate and nitrite metabolism by prominent fermentative bacteria in the rumen such as *Selenomonas ruminantium* as well as members belonging to *Butyrivibrio, Clostridium, Peptostreptococcus*, and *Propionibacterium* can readily and rapidly contribute enhanced nitrate reduction capabilities (Alaboudi, 1984; Iwamoto et al., 2002). Subsequent to induction of nitrate and nitrite reducing activity, these bacteria can be enriched in number via exposure to nitrate because of the greater energy yield from electron transport mediated phosphorylation of ADP to ATP or via achieval of more effective electron disposal (Hasan and Hall, 1975; Thauer et al., 1977; Moreno-Vivián et al., 1999). The contribution of propionibacteria to ruminal nitrate reduction is probably atypical, however, as most nitrate-reducing propionibacteria are denitrifiers that produce nitrous oxide as an end product (Kaspar, 1982). *Wolinella succinogenes* may be considered a specialist in that it is nonfermentative and exhibits highly active nitrate and nitrite reducing activity. Nitrate-utilizing *Veillenolla parvula* also contribute to ruminal nitrate and nitrite metabolism however the abundance of these populations appears to be dependent on nitrate concentrations (Asanuma et al., 2002; Iwamoto et al., 2002). A number of other nitrate-reducing bacteria, such as certain species of *Desulfovibrio* and members of family Enterobacteriaceae can inhabit the rumen, albeit at low abundance, and it is reasonable to suspect these bacteria could also be enriched during prolonged exposure to nitrate (Pfennig et al., 1981; Stewart, 1988). Certain members of Enterobacteriaceae, such as entertoxigenic and enterohemorrhagic *Escherichia coli* and numerous *Salmonella enterica* serovars, are important animal or foodborne pathogens and thus their enrichment due to nitrate feeding would be undesirable, although to our knowledge this has not yet been reported.

**Table 3:** **Summary of nitrate and nitrocompound toxicity**.

**Substrate**	**Source**	**Intoxication**	**Organisms involved**	**Transformation/metabolism**
Nitrate	Feeds	See nitrite	Many groups	Reduced to nitrite
Nitrite	Produced from nitrates in feeds	Methemoglobinemia	Many groups	Further broken down into ammonium
3-Nitro-1-propanol	*Astragulus* and many other plant species: hydrolysis of nitroglycosides in feed	Inhibits succinate dehydrogenase	*Denitrobacterium detoxificans, Coprococcus sp., Megasphaera elsdenii, Selenomonas ruminantium*	Metabolized to aminopropanol in the rumen and 3-nitro-1-propionate in the liver
3-Nitro-1-propionate	*Astragulus* and many other plant species: hydrolysis of nitroglycosides in feed	Inhibits succinate dehydrogenase	*Denitrobacterium detoxificans, Coprococcus sp., Megasphaera elsdenii, Selenomonas ruminantium*	Metabolized to β-alanine in the rumen which is futher metabolized
Nitroethane/nitroethanol	Synthetic	Unknown (possible respiratory toxicosis)	*Denitrobacterium detoxificans*	Metabolized to ethylamine/possibly to ethanolamine

Evidence for enrichment of nitrate-reducing bacterial populations also comes from studies of Alaboudi and Jones (1985), who reported >3-fold increases in rates of ruminal nitrate and nitrite metabolism in goats adapted to approximately 1.5 g nitrate (as KNO_3_) kg^−1^ body weight which coincided with a 3-fold increase in the proportion of nitrate-reducing bacteria. Unfortunately, they did not further characterize their isolated bacteria and the identity of nitrate-reducers was not reported (Alaboudi and Jones, 1985). More recently, Asanuma et al. (2015) reported 2.3–2.8-fold increases in nitrate and nitrite reducing activity following adaptation to a diet supplying approximately 0.18 g nitrate (as KNO_3_) kg^−1^ body weight per day to goats. Concomitant with this increase in nitrate and nitrite reducing activity were nearly equivalent increases in the relative abundance of *narG* and *nrfA* gene sequences specific for the nitrate reducing bacterium, *Selenomonas ruminantium*, as well this bacterium's 16S rRNA gene (Asanuma et al., 2015).

Conversely, Lin et al. (2013a) found no differences in abundance of *nar* or of 16S rRNA genes specific to *S. ruminantium* or to the less abundant nitrate-reducers *V. parvula* and *W. succinogenes* in rumen contents from nitrate nitrogen-fed steers (0.22–0.31 g nitrate kg^−1^ body weight, fed as KNO_3_) when compared to urea nitrogen-fed steers. Unfortunately, rates of nitrate and nitrite metabolism were not reported so comparison of abundance measurents to activity measurements is not possible. They did, however, observe increases in populations of the nitrate-reducers *Campylobacter fetus*, which was enriched in both liquid and solid fractions of ruminal contents, and *Mannheimia succiniciproducens*, which was enriched only in the liquid fraction collected from the rumen (Lin et al., 2013a). More recently, Zhao et al. (2015) proposed that *Campylobacter* and cyanobacteria were important nitrate-reducing taxa based on results from 16s rDNA sequencing. *Campylobacter fetus* is an important pathogen affecting ruminants and thus its enrichment would be undesirable. Conversely, *M. succiniciproducens* may be an attractive bacterium to enrich in the rumen because it has considerable potential fix carbon dioxide, via carboxylation of phospoenolpyruvate (Lee et al., 2006). Even after enrichment, however, *C. fetus* and *M. succiniciproducens* made up only a small proportion (< 0.1%) of the total population (Lin et al., 2013a). Moreover, the study of Lin et al. (2013a) was a cross-over design, however, with a 12 day wash-out period between nitrate-nitrogen and urea-nitrogen treatments which may have been insufficient to allow populations to re-achieve unperturbed densities. It was not stated if the animals had opportunity to physically contact one another during provision of the respective treatments, but if they had then induction or transfer of nitrate and nitrite reductive capacity could have occurred between groups of animals as has been as reported by Majak and Cheng (1984) and Cheng et al. (1985). In these earlier experiments, animals in treated groups received daily doses of nitrate intraruminally (0.1 g nitrate kg^−1^ body weight, salt unspecified) during the treatment period and were initially kept away from animals in the untreated control group (Majak and Cheng, 1984; Cheng et al., 1985). Then both groups were housed in adjacent pens while the treated group continued to receive the nitrate supplement. Despite receiving no nitrate, the control group of steers showed increased (80–200%) nitrate and nitrite reduction rates during the contact period. The authors hypothesized this transfer may have been mediated via horizontal gene transfer of plasmids containing nitrate reductase (Majak and Cheng, 1984; Cheng et al., 1985). However, this could also originate from other mobile genetic elements such as transposons or bacteriophages, oral transfer of microbes via animal licking, or some other signaling molecule that we are unaware of that would induce the upregulation of nitrate and nitrite reducing genes in the ruminal microbial population.

Exposure of ruminal populations of bacteria to nitrate not only selects for nitrate-reducing bacteria, but also acts as a selection mechanism for a different ecological makeup within the microbial community. Marais et al. (1988), using *in vitro* cultivation techniques, reported that nitrite accumulation resulting from reduction of added nitrate (approximately 26 g nitrate kg^−1^ dry matter; added as KNO_3_) decreased ruminal cellulolytic activity. They concluded that the decrease in cellulolytic activity was a result of 64, 25, and 57% decreases in numbers of cellulolytic, xylanolytic and total viable bacteria, respectively, as determined via viable cell count on selective media. Others also have observed toxic effects of nitrite, produced as an intermediate during the reduction of nitrate, on populations of cellulolytic bacteria as well as on other microbial populations including methanogens (Iwamoto et al., 2002; Zhou et al., 2011, 2012; Asanuma et al., 2015). The inhibition of cellulolytic organisms may explain the decreases in dry matter intake sometimes observed in animals feed nitrate-supplemented diets (Newbold et al., 2014; Lee et al., 2015a,b). It is recognized that decreased cellulolysis can decrease rates and extent of neutral detergent fiber digestion thus increasing rumen retention time of a forage and negatively affective rumen fill, both which can cause decreased dry matter intake (Allen, 2000). It is well known that nitrate and nitrite additions cause a shift in volatile fatty acid concentration, sometimes disproportionately against branched-chain volatile fatty acids essential for certain bacterial populations, and this has been suggested as a reason for decreases observed in total bacterial populations and particularly cellulolytic bacteria (Allison and Reddy, 1984) but this was discounted by Marais et al. (1988).

In the case of methanogens, the reduction of nitrate preferentially consumes electrons at the expense of methanogenesis. However, methanogens also appear to be particularly sensitive to the toxic effects of nitrite, with 50% inhibition in cell growth occurring with as little as 0.5 mM nitrite (Iwamoto et al., 2002). Asanuma et al. (2015) similarly found methanogens, as well as total populations of rumen bacteria, protozoa and fungi to be greatly decreased *in vivo* after goats were fed a high nitrate diet (5.4 g nitrateday^−1^, as KNO_3_) for 2 weeks. This total community depression may result from the oxidizing nature of nitrite as it relates to its antimicrobial properties, attributed to inactivation or inhibition of sulfur containing constituents involved in energy metabolism, DNA replication or maintenance of cell wall integrity (Marais et al., 1988; Cammack et al., 1999).

The high reactivity of nitrite could also disrupt the low E°/mV within the rumen as evidenced in the study of Jamieson (1959), who found that sheep intraruminally dosed with 25 g nitrate (as KNO_3_) had a higher *E*_h_ value pre-dose than 2 h post dose (−225 vs. −70 mV, respectively). As reported by Kalachniuk et al. (1978) and discussed by Zhou et al. (2012), an increase in reduction potential (*E*_h_) has been reported to be inhibitory to some rumen bacteria, notably *S. ruminantium, Bacteroides amylophilus, Fibrobacter* (*Bacteroides*) *succinogenes* and *Streptococcus bovis*. More recently, however, an increased *E*_h_ was found to be not particularly inhibitory per se to these same bacterial species (Marounek and Wallace, 1984), but it could potentially perturb thermodynamic control of important oxidation/reduction reactions such as those involved in electron transfer. For instance, Marais et al. (1988) reported that nitrate-caused inhibition of forage digestibility could not be overcome by using the reducing agent cysteine to decrease the nitrite-caused increase in culture *E*_h_. Thus, they proposed a direct effect of nitrite on inhibiting bacterial growth, and while they at the time suggested this inhibition appeared to be most potent against bacteria with electron transport linked phosphorylation capabilities, more recent evidence suggest the toxic effect may not be so specific. In the study of Asanuma et al. (2015) for instance, they found that populations of major the cellulolytic bacteria *F. succinogenes, Ruminococcus flavefaciens*, which contain electron transport capabilities, as well *Ruminococus albus*, which does not contain electron transport capabilities, all decreased in the rumen of goats fed nitrate. Zhou et al. (2012) also reported reductions in abundance of *F. succinogenes, R. flavefaciens*, and *R. albus* as well as in archaeal, but not total bacterial populations when measured by real-time PCR during *in vitro* incubations of mixed populations of ruminal microbes with =12 mM sodium nitrate. They further found evidence of adaption or acquisition of tolerance by populations of *R. albus* as well as *F. succinogenes*, but not *R. flavefaciens* or the archaeal populations following up to 6 consecutive cultures with 12 mM added sodium nitrate (Zhou et al., 2012). Broad activity of nitrite was found in the studies of Iwamoto et al. (2002), who tested 15 different bacterial species and found that growth of all except four were inhibited by concentrations of 3–5 mM nitrite, with many being producers of hydrogen, formate or lactate that can contribute reductants for nitrate and nitrite reduction or methanogenesis (Russell and Rychlik, 2001). Decreases in populations of hydrogen, formate or lactate-producing microbes could potentially limit the availability of reductant for methanogenesis or nitrate respiration. However, Asanuma et al. (2015) observed significant increases in genetic abundance of the highly efficient sugar-fermenting bacterium, *S. bovis*. It is reasonable to speculate that the sugar-fermentation by *S. bovis* may contribute a pool of electron donating substrates for the nitrate-reducing population thus potentally compensating for the decreased reductant that would be expected by inhibition of the hydrogen, formate or lactate-producing microbes observed by Iwamoto et al. (2002).

In direct opposition to the findings discussed above, Zhao et al. (2015) found that added nitrate *in vivo* (1–2% dry matter, nitrate type unknown) was associated with an increase in many cellulolytic bacterial species including *R. flavefaciens, R. albus*, and *F. succinogenes*. The authors attributed this selection to nitrate-caused increase production and thus availability of branch chain fatty acids required by these bacteria. The authors did not specifically discuss why branched chain fatty acids were increased but it is reasonable to speculate that this may have occurred due to changes in microbial diversity in the rumen population.

The protozoa, which reduce nitrate to ammonia for assimilatory purposes, play an unclear role in the total nitrate metabolism in the rumen. The protozoal fractions showed similar rates of nitrate reduction with less nitrite accumulation without any adaptation period as compared to the whole rumen fraction or bacterial fraction (Yoshida et al., 1982; Iwamoto et al., 2001; Lin et al., 2011). In contrast, Allison and Reddy (1984) reported that the nitrate and nitrite reducing activity was not found in their protozoal fraction. Moreover, they also reported that the nitrate reducing activity in rumen contents was membrane bound and was inhibited by azide and hydroxyl quinolone-N-oxide, each inhibitors of electron carrier mediated respiration. The absence of nitrate-reducing activity when dissimilatory (respiratory) nitrate reduction was inhibited suggests that the assimilatory nitrate-reducing pathway, such as that by protozoa, was inoperative or contributed little to overall nitrate reduction. Future studies are needed to detect changes in the protozoal populations in the rumen from nitrate-fed ruminants as well as additional research exploring their potential, if any, to enhance nitrite removal and reduce methane production.

## Other effects of feeding nitrate

Additions of nitrate to *in vitro* incubations of mixed populations of ruminal microbes have generally resulted in decreased production of methane and propionate while sometimes increasing production of total volatile fatty acids; however, this varies considerably depending on experimental conditions (Bozic et al., 2009; Shi et al., 2012; Zhou et al., 2012; Patra and Yu, 2014). The decreased production of more reduced fatty acids reflects a lesser need for this route of electron disposal because electrons are instead consumed for the reduction of nitrate to nitrite and ultimately to ammonia (Sutherland, 1977; Russell, 2002). Increased acetate production at the expense of butyrate can sometimes occur due to nitrate supplementation (Farra and Satter, 1971; Anderson and Rasmussen, 1998) and this could be due to the stimulation of more thermodynamically favorable pathways for electron disposal (Ungerfeld and Kohn, 2006).

The effects of nitrate feeding ultimately depend on nitrate concentration and its availability within the rumen which is not only affected by the amount fed but also by the type or source of nitrate fed which can markedly affect the ruminal availability of the free nitrate ion. Consequently, *in vivo* additions of nitrate have also resulted in variable effects on volatile fatty acid production and methane emissions as well as on methemoglobin development in studies measuring these responses (Table 4). For instance, encapsulated nitrate supplements used in the studies of Lee et al. (2015a,b) would be expected to release nitrate more slowly than the salt forms of nitrate, which themselves can differ in nitrate availability thus affecting their methane reducing potential and their toxicity. In studies using calcium nitrate, amounts consumed ranged from 0.26 to 0.94 g Ca(NO_3_)_2_kg^−1^ body weight, an amount sufficient to cause toxicity if an equivalent dose of sodium or potassium nitrate were fed (van Zijderveld et al., 2010, 2011; Hulshof et al., 2012; Li et al., 2012; de Raphélis-Soissan et al., 2014; El-Zaiat et al., 2014; Velazco et al., 2014). Likewise, calcium nitratewas the least effective at decreasing methane, 3.5% reduction when expressed as mmol^−1^ nitratekg^−1^ body weight, as compared to sodium and potassium nitrate which caused a 14.5 and 9.6% decrease in methane per mmol nitratekg^−1^ body weight respectively (Takahashi and Young, 1991; Sar et al., 2004, 2006; Nolan et al., 2010; van Zijderveld et al., 2010, 2011; Hulshof et al., 2012; Leng et al., 2012; Li et al., 2012; de Raphélis-Soissan et al., 2014; El-Zaiat et al., 2014; Velazco et al., 2014; Asanuma et al., 2015). Similarly, Phuong (2012) found that 24% less NaNO_3_ and 12% less KNO_3_ as compared to Ca(NO_3_)_2_ resulted in the same amount of methane production. This is most likely due to differences in solubility. Calcium has low solubility at pH above 5.5 and since the rumen pH seldom declines to below 5.5 it is likely that a significant portion of the calcium nitrate is unsolubilized and thus unavailable to exert the full effect of the nitrate. Additionally, the cation themselves (K, Na, and Ca) have important roles in the rumen including motility, osmolality, pH, acid base balance, and are known to influence the dry matter intake. As many of these nitrate salt feeding trials are feeding at level in excess of 2% of intake, there is the possibility that the accompanying cations may alter the microbiome and the animal physiology. Iso-cation diets controls may be necessary in future studies. Overall, it would be prudent to develop standards for nitrate salt feeding with stipulations for salt type, age of the animal, adaptation status, and previous exposure.

**Table 4:** **Effects of nitrate supplementation to ruminants on volatile fatty acid production, metHb, and methane reduction *in vivo***.

**Author**	**Nitrate source^a^**	**Effects on volatile fatty acids produced (*P* < 0.05)**				**Administered**	**Animal**	**Max MetHb (%)**	**Methane effect (%)**	**% Dry matter intake**	**Highest amount supplemented^b^**
		**Total acids**	**Acetate**	**Propionate**	**Butyrate**						
Alaboudi and Jones, 1985	KNO_3_	No effect	Increased	Decreased	Decreased	Top dressed	Sheep	2.0	NM^c^	0.05	0.02480
Asanuma et al., 2015	KNO_3_	Decreased	Decreased	Decreased	Decreased	Top dressed	Goats	NM	−89	NM	0.00310
de Raphélis-Soissan et al., 2014	Ca(NO_3_)_2_	No effect	Increased	Decreased	NM	Top dressed	Sheep	45.0	19	2.0	0.00767
El-Zaiat et al., 2014	Ca(NO_3_)_2_:NH_3_NO_3_	Increased	Increased	No effect	Increased	Encapsulated	Lambs	1.0	−33	4.5	0.02110
Farra and Satter, 1971	KNO_3_:NaNO_3_	No effect	Increased	Decreased	Decreased	Top dressed	Sheep	2.0	NM	6.0	0.00510
Hulshof et al., 2012	Ca(NO_3_)_2_	No effect	No effect	No effect	No effect	Top dressed	Beef	NM	−32	2.5	0.00600
Lee et al., 2015a,b	Ca(NO_3_)_2_	No effect	No effect	No effect	No effect	Encapsulated	Beef	< 2.0	18	2.5	0.00844
Leng et al., 2012	KNO_3_	NM	NM	NM	NM	Top dressed	Beef	< 1.0	−29	6.0	0.01020
Li et al., 2012	Ca(NO_3_)_2_	No effect	Increased	No effect	Decreased	Top dressed	Sheep	< 1.0	−35	3.0	0.01150
Lund et al., 2014	Ca(NO_3_)_2_	No effect	No effect	No effect	No effect	Top dressed	Dairy^d^	NM	−31	0.7	0.00120
Newbold et al., 2014	Ca(NO_3_)_2_	NM	NM	NM	NM	Top dressed	Beef	>20	−30	3.0	0.00266
Nolan et al., 2010	KNO_3_	Decreased	Increased	Decreased	Decreased	Top dressed	Sheep	< 1.0	−23	4.0	0.00680
Pal et al., 2014	KNO_3_	No effect	No effect	Decreased	No effect	Top dressed	Sheep^d^	NM	NM	2.0	0.00600
Sar et al., 2004	NaNO_3_	Increased	Increased	Decreased	Decreased	Via cannula	Sheep	18.4	−50	n/a^c^	0.00580
Sar et al., 2005	NaNO_3_	No effect	Increased	No effect	Decreased	Via cannula	Sheep	2.0	−50	n/a	0.00580
Takahashi and Young, 1991	NaNO_3_	NM	NM	NM	NM	Via cannula	Sheep^d^	20.0	−86	n/a	0.00188
van Zijderveld et al., 2010	Ca(NO_3_)_2_	No effect	No effect	No effect	No effect	Top dressed	Lambs	7.0	−32	2.6	0.00805
van Zijderveld et al., 2011	Ca(NO_3_)_3_	NM	NM	NM	NM	Top dressed	Dairy	4.7	−16	1.0	0.00366
Velazco et al., 2014	Ca(NO_3_)_2_	NM	NM	NM	NM	Top dressed	Beef	3.0	−17	2.6	0.00960

a *KNO_3_, potassium nitrate; Ca(NO_3_)_2_, calcium nitrate; Ca(NO_3_)_2_:NH_4_NO_3_, 50:50 calcium nitrate:ammonium nitrate; KNO_3_:NaNO_3_, 50:50 potassium nitrate:sodium nitrate; NaNO_3_, sodium nitrate*.

b *mol/kg body weight*.

c *NM, not measured; n/a, not applicable*.

d *Animal were not previously adapted to nitrate additions*.

In global warming potential (GWP), carbon dioxide is 1, methane is 21, and nitrous oxide is 298 over a 100-year time scale. Therefore, even a small increase in nitrous oxide from nitrate reduction could have large effects on GWP. For example, nitrous oxide emission from sheep was higher when fed nitrate (calcinite at 0.625 g kg^−1^ body weight), which in turn lowered the net benefit of methane mitigation on global warming potential by 18% despite the nitrous oxide being only 0.3% of added nitrate (de Raphélis-Soissan et al., 2014). Likewise, Petersen et al. (2015) found a 28–23% reduction in overall decrease in global warming mitigation effect and Neumeier et al. (2014) found no net reduction in greenhouse gases when considering the increase in nitrous oxide from feeding nitrate.

Considering the cost and risk to the producers of feeding nitrate, the decrease of greenhouse gases production may not justify the usage of nitrate feeding as a methane mitigation strategy. Toxicity aside, it has been suggested that the limiting factors for the adoption of supplemental-nitrate feeding in beef production are financial in nature (Callaghan et al., 2014).

## Nitrocompound metabolism within the rumen

Unlike nitrate and nitrite metabolism, less is known about the mechanisms of nitrocompound metabolism by ruminal microbes. Nishino et al. (2010) isolated 3-nitro-1-propionate-degrading species of strictly aerobic *Cupriavidus* and *Pseudomonas* bacteria from soil and water. Considering, however, that their degradation of 3-nitro-1-propionate proceeded to yield to propionate-3-nitronate and ultimately malonic semialdehyde, nitrate, nitrite, and hydrogen peroxide, it is unlikely these aerobic bacteria contribute to nitrocompound degradation in the anaerobic rumen. In the rumen, the naturally occurring 3-nitro-1-propionic acid and 3-nitro-1-propanol are first hydrolyzed from their glucose conjugates by microbial esterase or β-glycosidase activity thereby liberating the aglycones from their respective esters or ether glycosides (Table 3), the latter which are rather innocuous (Anderson et al., 2005). Once liberated, 3-nitro-1-propionic acid and 3-nitro-1-propanol are available to be absorbed or further metabolized by bacteria in the rumen. The nitroacid is absorbed less rapidly but metabolized more rapidly than the nitroalcohol which thus explains why 3-nitro-1-propionic acid is less toxic than 3-nitro-1-propanol to ruminants.

Presently, the ruminal bacterium *Denitrobacterium detoxificans* is the only known anaerobe to exhibit appreciable nitroalkane-reducing activity (Anderson et al., 1996, 1997, 2000). This bacterium conserves energy for growth exclusively via anaerobic respiration, oxidizing hydrogen, formate or lactate in the reduction of nitrate to ammonia and the reduction of 3-nitro-1-propionic acid, 3-nitro-1-propanol, and nitroethane to β-alanine, 3-amino-1-propanol and aminoethane, respectively (Anderson et al., 1993, 1997, 2000). β-alanine was rapidly metabolized to unknown products in mixed populations of ruminal microbes but 3-amino-1-propanol appeared to be a terminal product (Anderson et al., 1993). Early research by Looper et al. (1959) indicated that β-alanine was appreciably deaminated in the rumen but little other information is available on its fate as an endproduct. The reduction of the nitroalkanes to their amines is presumed to consume six moles electrons per mol of amino group reduced and is based on the stoichiometry reported for the reduction of nitroethanol to ethanolamine (Angermaier and Simon, 1983). *Denitrobacterium detoxificans* can also grow on dimethyl sulfoxide and trimethyl amine oxide, reducing these acceptors to dimethylsulfide and trimethyl amine, and can grow, albeit less readily, on nitroethanol, 2-nitro-4-butanol, 1-nitropropane and 2-nitro-1-propanol (Anderson et al., 2000). The fate of these later four substrates has not been investigated. With respect to these nitrocompounds, all except 2-nitro-4-butanol and 1-nitropropane have been tested and found to be potent inhibitors of ruminal methanogenesis (Anderson and Rasmussen, 1998; Anderson et al., 2003, 2008, 2010). Evidence indicates that *D. detoxificans* can effectively contribute to detoxification of 3-nitro-1-propionic acid, 3-nitro-1-propanol, which exist as phytotoxins in certain leguminous plants, notably milkvetchs belonging to the genera *Astragalus* and *Coronilla* (Anderson et al., 2005). However, with little known about the physiology and nutritional requirements more research is needed to make growing this bacterium practical for large scale applications.

More recently, chemically synthesized dimethyl-2-nitroglutarate and 2-nitro-methyl-propionate have also been tested and found to be similarly potent anti-methanogenic compounds (Anderson et al., 2010) as has ethyl nitroacetate (data not shown); however, the fate of these three nitrocompounds within incubations of mixed populations of rumen bacteria has not been determined.

From a thermodynamic perspective, Gibbs free energy values for the reduction of the nitrocompounds to their respective amines have not been determined. However, experimentally, nitrate was preferentially reduced by mixed populations of bovine ruminal microbes compared to 3-nitro-1-propionate or 3-nitro-1-propanol when the compounds were incubated together (Anderson et al., 1997; Zhang et al., 2014). This suggests that the reduction of nitrate may be energetically more favorable than the reduction of the nitrocompounds although the contribution of other mechanisms, such as the presence of more active nitrate-reducing enzymes cannot be excluded.

The two different mechanisms of action of these nitroalkanes, primarily direct inhibition of methanogenesis and secondarily acting as an alternative electron acceptor, are not necessarily incompatible as the process that directly inhibits methanogenesis may promote the redirection of electrons and thereby facilitate the reduction of the nitroalkanes. Subsequent *in vitro* studies have reported significant decreases in methane production by ruminal microbial populations treated with 4–13 μmol nitroethane/mL with modest affects on volatile fatty acid production and with recovery of electrons, expressed as hydrogen recoveries, accounting for 37–52% of the decrease in methane production (Gutierrez-Bañuelos et al., 2008; Bozic et al., 2009). The fate of the remaining hydrogen was undetermined but the authors speculated that other unmeasured sinks such as ethanol, formate or anabolic processes, such as those contributing to cell growth could be functional sinks (Gutierrez-Bañuelos et al., 2008; Bozic et al., 2009) as could reduced products of nitrocompound metabolism or the production of extracellular or intracellular polysaccharide production. Several other studies using a variety of other short chain nitrocompound have supported these findings, with only a few exceptions, the latter possibly resulting from a lesser or even an inability to dispose of electrons onto the nitrocompounds or attributable to differences in cultural conditions (Table 5). For instance, dimethyl-2-nitroglutarate and 2-nitro-methyl-propionate were found to effectively decrease ruminal methane production during an *in vitro* batch culture with ruminal microbes (Anderson et al., 2010). However, while it was anticipated that hydrolysis of the methyl esters and reduction of the nitrocompounds would yield methionine and alanine as potential endproducts of use to the host, no such evidence was obtained (Anderson et al., 2010). The microbial population had no prior exposure to these nitrocompounds; therefore it would be worthwhile to determine if adapted populations may be able to metabolize the nitrocompounds (Anderson et al., 2010).

**Table 5:** **Effects of nitrocompound supplementation on methane and volatile fatty acid production during *in vitro* rumen incubations**.

**Nitrocompound**	**Author**	**Concentration (μmol/mL)^a^**	**Initial culture gas phase**	**Methane reduction**	**Effects on volatile fatty acids produced (*P* < 0.05)**			
					**Total acids**	**Acetate**	**Propionate**	**Butyrate**
3-Nitro-1-propionate	Anderson and Rasmussen, 1998	5–20	50:50 H_2_:CO_2_	19–69%	Increased	No effect	Increased	Increased
Nitroethane	Gutierrez-Bañuelos et al., 2008	4–9	50:50 H_2_:CO_2_	89–80%	No effect	No effect	No effect	Increased
Nitroethane	Bozic et al., 2009	13	50:50 H_2_:CO_2_	99%	Decreased	Increased	Decreased	Increased
Nitroethane	Anderson et al., 2003	2–24	CO_2_	58–95%	Variable	Variable	Variable	Variable
Nitroethanol	Anderson et al., 2003	12	CO_2_	95%	No effect	No effect	No effect	No effect
2-Nitro-1-propanol	Anderson et al., 2003	12	CO_2_	91%	No effect	No effect	No effect	No effect
Nitroethane	Anderson et al., 2010	3–12	CO_2_	94–99%	No effect	No effect	Increased	Increased
Dimethyl-2-nitroglutarate	Anderson et al., 2010	3–12	CO_2_	92–97%	No effect	No effect	No effect	No effect
2-Nitro-methyl-propionate	Anderson et al., 2010	3–12	CO_2_	98%	No effect	No effect	No effect	No effect
Nitroethane	Zhou et al., 2011	12	10:15:85 H_2_:CO_2_:N_2_	None	No effect	No effect	No effect	No effect
Nitroethanol	Zhou et al., 2011	12	10:15:85 H_2_:CO_2_:N_2_	99%	Decreased	Decreased	Decreased	Decreased

a *Concentrations have been rounded to nearest whole number*.

With respect to *in vivo* studies, doses of 24 and 72 mg nitroethane and 40 and 120 mg 2-nitro-1-propanol (per kg body weight^−1^ day^−1^) resulted in methane-reducing activity being 37–69% lower than that in untreated controls, with nitroethane being more potent than 2-nitro-1-propanol (Anderson et al., 2006a). Methane-producing activity was measured via *in vitro* incubation of freshly collected rumen contents with excess methanogenic substrate concentrations and thus conceptually represents an indirect measure of numbers of methanogens, assuming their numbers are correlated with quantities of methane-producing enzymes. When administered to cattle at doses of 30–120 mg nitroethane kg body weight^−1^ day^−1^, methane-producing activity was decreased by 6 to as much as 44% when compared to controls, although methane production *in vivo*, measured using the sulfur hexafluoride technique, were decreased by no more than 22% (Gutierrez-Bañuelos et al., 2007; Brown et al., 2011). Oral administration of 2-nitro-1-propanol decreased ruminal acetate concentrations from that of controls by about 15% and the high nitroethane dose (120 mg kg body weight^−1^ day^−1^) decreased rumen acetate concentrations by about 24% in the fed steers of the study of Gutierrez-Bañuelos et al. (2007). When measured, rates of ruminal nitroethane-metabolizing activity were increased more than 30% but these increases were not always significant (Gutierrez-Bañuelos et al., 2007; Brown et al., 2011) thus suggesting that onset and duration of enrichment of competent nitroethane-metabolizing bacteria is highly variable between animals and possibly reflects de-enrichment in steers fed the lower doses due to depletion of ruminal nitroethane concentrations. Similarly, there was evidence of ruminal adaptation of the ruminal population to the lower but not the higher nitroethane dose, with methane-producing activity approaching control levels by 7 days of treatment in steers fed 80 mg nitroethane kg body weight^−1^ (Gutierrez-Bañuelos et al., 2007). This too possibly reflects a depletion of efficacious concentrations of nitroethane in rumen contents of low-dosed steers due to consumption by nitroethane-metabolizing bacteria. In the steers fed 120 mg nitroethane kg body weight^−1^ day^−1^, for instance, decreases in methane-producing activity persisted to the end of the 15 day administration. Thus, it is likely the higher dose not only allowed consumption of more reductant than the lower dose but also retained sufficient residual nitroethane concentration to maintain inhibitory pressure on methanogens and sustain growth of the nitroethane-metabolizing bacteria.

More recently, other researchers have observed 75–95% decreases in methane production during *in vitro* incubation of mixed populations of ruminal microbes treated with 0.05–0.66 μmol/mL ethyl-3-nitrooxy propionate or 3-nitrooxypropanol that suggests a greater potency of these more oxidized nitrocompounds (Martínez-Fernández et al., 2014; Romero-Pérez et al., 2015a). In the Rusitec study of Romero-Pérez et al. (2015a), hydrogen recovery in volatile fatty acid products was increased approximately 13–14% due to 3-nitrooxypropanol treatment and hydrogen accumulations were increased more than 3-fold yet recovery of all of the reductant spared from methanogenesis was not achieved. These authors also speculated that formate could possibly be an unmeasured hydrogen sink, but the potential metabolism of the nitroxy compounds to reduced products has not been discussed. A number of studies have examined the effect of 3-nitrooxypropanol on ruminal methane emissions from dairy or beef cattle and results have yielded positive results, with methane emissions, based on dry matter intake, being decreased from controls by 6 to nearly 60% (Haisan et al., 2014; Reynolds et al., 2014; Romero-Perez et al., 2014, 2015b; Hristov et al., 2015). Negative effects on animal performance, if observed, were modest. Variability in efficacy between studies possibly reflects differences in administration protocols (intraruminal vs. in feed) and methane measurement techniques. In the study of Hristov et al. (2015), as much as a 64-fold increase in hydrogen emissions was observed in 3-nitrooxypropanol-treated cattle, which was still only about 3% of the hydrogen spared from methanogenesis.

Few studies, as of yet, have determined the microbial response to nitrocompounds. At approximately 4 mM concentration, the naturally occurring nitrocompounds, 3-nitro-1-propionate and 3-nitro-1-propanol, were found to be modestly inhibitory to total culturable anaerobes from the bovine rumen, but the inhibited populations were not characterized (Anderson et al., 1993). These compounds as well as nitroethane, nitroethanol and 2-nitro-1-propanol are also inhibitory to uric acid degrading bacteria of poultry origin (Kim et al., 2005, 2009). Additionally, nitrocompounds, similarly to nitrates, inhibit the growth of many foodborne pathogens including *Listeria, Salmonella*, and *Campylobacter* and enteropathogenic *E. coli* (Jung et al., 2003, 2004a,b; Anderson et al., 2006b; Dimitrijevic et al., 2006), a use that has been patented (Anderson et al., 2007).

## Factors that influence nitrate and nitrocompound metabolism

There is a significant amount of variation between animals with regard to their response to nitrate addition. Part of this response is due to the rumen ecosystem. The largest influencer would be the endogenous microbial populations and their enzymatic capacity. However, there are many other factors that influence the rate of reduction of nitrate, nitrite, and nitrocompounds.

First, the availability and amount of substrate supplying electrons used for the reductive nitrate reactions including carbohydrates, ethanol, mannitol, glycine, malate, citrate, lactate, succinate, pyruvate and formate (Lewis, 1950, 1951; Jones, 1972). Fatty acids, acetic, propionic, and butyric are not electron donors for nitrate reduction under this metabolic process. Thus, supplementing or provision of diets rich with these electron donors can potentially enhance nitrate reduction to ammonia, thereby further enhancing the decrease in methane and reducing the chances of nitrate poisoning as the animal will be exposed to less of the toxic intermediate nitrite which converts hemoglobin to methemoglobin also known as methemoglobinemia (Takahashi et al., 1980).

Dietary interventions such as high starch and cereal grains have been proposed to help mitigate nitrate toxicity (Burrows et al., 1987; Hibberd et al., 1994) purportedly by stimulating the metabolism of ruminal microorganisms thereby promoting production and subsequent availability of electron-donating substrates for nitrate and nitrite metabolism (Lewis, 1950, 1951; Allison and Reddy, 1984). High starch diets, within a pH range of about 6.6–6.8, have been shown to increase hydrogen production by mixed populations of ruminal microbes *in vitro* (Lin et al., 2013b). However, high starch diets are also well established to decrease rumen pH, which could potentially create an environment inhibitory to ruminal nitrate and nitrite metabolism. While enzymatic analysis indicates nitrate and nitrite reductases work optimally at pH of 6.5 or 5.6, respectively (Lewis, 1951; Tillman et al., 1965), nitrate- and nitrite-reducing activity within mixed populations of ruminal microbes was more rapid at neutral pH than at pH 6.0 or lower (Iwamoto et al., 2001). The inhibitory activity of the lower pH on nitrate- and nitrite-reducing activity within mixed rumen populations was attributed to lower availability of electron-donating substrates such as hydrogen, formate, or lactate resulting from an inhibition of fermentation caused by the low pH (Iwamoto et al., 2001). This would suggest that nitrate supplemented to concentrate fed-cattle may be ineffective or at least less effective in decreasing ruminal methane emissions than when supplemented to forage fed cattle. From a methane mitigation perspective, this may be of little consequence as the low ruminal pH in high concentrate-fed ruminants also inhibits methane production (Van Kessel and Russell, 1996; Lana et al., 1998; Russell, 1998) and thus concentrate-fed ruminants emit significantly less methane than do forage-fed cattle (Johnson and Johnson, 1995). From a toxicity perspective, however, it seems likely that supplementing nitrate to animals expected to have low rumen pH may increase risk for nitrite accumulation and subsequent toxicosis due to methemoglobinemia.

The next consideration is adaptation of the rumen microbial population to high nitrate diets. As has been alluded to earlier, the ruminal microbial population can be selected for increased nitrate- and nitrite-reducing activity by gradual feeding of increasing amounts of nitrate. However, little is known as to what might happen to the microbial population if an animal is de-adapted and selective pressure is removed. For instance, it is reasonable to suspect that should an animal go off feed and refuse its meals for a certain period of time a disbalance between rates of nitrate reduction and nitrite reduction may occur, which if favoring high nitrite accumulation may place the animal at greater risk to nitrite intoxication. Establishing or dosing the animal with a population of probiotic bacteria capable of reducing nitrite to non-toxic forms may also reduce the concentration of nitrite in the rumen. Currently, there is one patent, which is sold as a direct-fed microbial additive for the prevention of nitrite toxicity in ruminants fed nitrate. It employs a denitrifying strain of *P. acidipropionici*; however, its nitrate and especially its nitrite reductive capacity have not been proven to remediate nitrate toxicity nor enhance methane reduction (de Raphélis-Soissan et al., 2014). There is also a genetically modified *E. coli* strain that has been developed with enhanced nitrite utilization that has been proven to work *in vitro* and *in vivo* (Sar et al., 2005, 2006). However, its status as a GMO and as a strain of a pathogenic genus may create a barrier too large for its usage in production.

Adaptation can occur at the animal, or systemic, level as well. Ruminants increase the amount of hemoglobin, red blood cells, and blood volume, thereby increasing the capacity to deliver oxygen and counter the effect of increasing methemoglobin. This process occurs over months (Jainudeen et al., 1964). In addition, NADPH reductase will convert methemoglobin back to hemoglobin (Hibberd et al., 1994). It is reasonable to hypothesize that animals adapted to nitrate have an elevated concentration of NADH reductase, but this has not been documented.

The rate of nitrate reduction also appears to be influenced by sulfur availability and concentrations. The reduction of sulfate to sulphite and then sulfide is less energetically favorable than nitrate to nitrite to ammonia. Opportunistically the enzymes for each reaction generally work for both reductive processes due to the molecule's similarity in structure and charge (Thauer et al., 1977). In addition, sulfur stimulates the growth of sulfide-reducing bacteria. Therefore, the addition of sulfur should hypothetically increase the amount of nitrate and nitrite reduced and the speed of the reaction (Leng, 2008). Experimentally, L-cysteine, which is rich in sulfur, in conjunction with nitrate has been shown to suppress the formation of methemoglobin (Takahashi et al., 1998).

Less is known regarding factors affecting nitrocompound metabolism within the rumen, although protein supplementation enhances *in vivo* rates of ruminal 3-nitro-1-propanol metabolism in cattle (Majak, 1992) and the reducing substrates, hydrogen gas and formate, are known to be stimulatory to reduction of the nitrocompounds *in vitro* (Anderson et al., 1993). Moreover, supplementing ruminal populations with ferrous and sulfide ions markedly increased rates of 3-nitro-1-propionic acid and 3-nitro-1-propanol metabolism (Anderson et al., 1993), but had little if any effect on the metabolism of these nitrocompounds in populations of equine cecal microbes (Zhang et al., 2014). Mechanistically, the ferrous and sulfide ion additions were thought to promote ferredoxin-hydrogenase mediated electron transfer reactions contributing to the reduction of the nitrocompounds.

## Conclusions

Nitrate and nitrite reduction to ammonia in the rumen is a more thermodynamically favorable reaction than the formation of methane with carbon dioxide as an electron acceptor. The effectiveness and risks of toxicity of this strategy are dependent on the nitrate salt type with sodium nitrate being the most biologically available and calcium the least in the rumen environment. A portion, albeit small, of the introducted nitrate appears to be metabolized to nitrous oxide either via dissimilatory nitrate reduction or more likely via incomplete denitrification, which may lessen its net greenhouse gas mitigation. A variety of certain short chain nitrocompounds as well as some nitro-oxy compounds have also been shown to decrease ruminal methane production. The naturally occurring nitrocompound, 3-nitro-1-propionate, is known to be metabolized by ruminal microbes to β-alanine, a non-essential amino acid that may be metabolized by the host and potentially used as a source of carbon, nitrogen and energy making it an attractive candidate. Safe and successful feeding of supplemental nitrate and nitrocompounds requires careful adaptation of the ruminal microbes to prevent risks of toxic intermediates and this practice could benefit via concurrent feeding of appropriate nitrite-reducing or nitrocompound degrading probiotic bacteria. Additionally, combination feeding of 3-nitro-1-propionate, or another appropriate nitrocompound, with subtoxic amounts of nitrate may yield synergistic advantages in inhibition of rumen methanogensis and electron capture in non-methane products.

More research is needed, however, on the pathways involved in nitrate and nitrocompound metabolism, the organisms involved, and the regulation of their enzyme activities in order to mitigate concerns that persist over the risks of toxicities and realize the full potential of these methane-decreasing strategies.

## Author contributions

EL: nitrate section and editing, RA: nitrocompound and editing, WP: editing, DN: supervision.

### Conflict of interest statement

The authors declare that the research was conducted in the absence of any commercial or financial relationships that could be construed as a potential conflict of interest.

## References

[B1] Alaboudi A. R. (1984). Microbiological Studies of Nitrate and Nitrite Reduction in the Ovine Rumen. PhD dissertation, University of Saskatchewan, Saskatoon.

[B2] Alaboudi A. R. Jones G. A. (1985). Effect of acclimation to high nitrate intakes on some rumen fermentation parameters in sheep. Can. J. Anim. Sci. 65, 841–849. 10.4141/cjas85-099

[B3] Allen M. S. (2000). Effect of diet on short-term regulation of feed intake by lactating dairy cattle. J. Dairy Sci. 83, 1598–1624. 10.3168/jds.S0022-0302(00)75030-2 10908065

[B4] Allison M. J. Reddy C. A. (1984). Adaptations of gastrointestinal bacteria in response to changes in dietary oxalate and nitrate, in Current Perspectives in Microbial Ecology, Proc. 3rd Int. Symp. on Microbial Ecology, eds Klug M. J. Reddy C. A. (Washington, DC: American Society for Microbiology), 248–256. Available online at: http://agris.fao.org/agris-search/search.do?recordID=US8605628 (Accessed July 20, 2015).

[B5] Anderson R. C. Buckley S. A. Kubena L. F. Stanker L. H. Harvey R. B. Nisbet D. J. (2000). Bactericidal effect of sodium chlorate on *Escherichia coli* O157: H7 and *Salmonella* Typhimurium DT104 in rumen contents *in vitro*. J. Food Prot. 63, 1038–1042. 10945577 10.4315/0362-028x-63.8.1038

[B6] Anderson R. C. Callaway T. R. Van Kessel J. A. S. Jung Y. S. Edrington T. S. Nisbet D. J. (2003). Effect of select nitrocompounds on ruminal fermentation; an initial look at their potential to reduce economic and environmental costs associated with ruminal methanogenesis. Bioresour. Technol. 90, 59–63. 10.1016/S0960-8524(03)00086-5 12835058

[B7] Anderson R. C. Carstens G. E. Miller R. K. Callaway T. R. Schultz C. L. Edrington T. S.. (2006a). Effect of oral nitroethane and 2-nitropropanol administration on methane-producing activity and volatile fatty acid production in the ovine rumen. Bioresour. Technol. 97, 2421–2426 10.1016/j.biortech.2005.10.013 16303299

[B8] Anderson R. C. Huwe J. K. Smith D. J. Stanton T. B. Krueger N. A. Callaway T. R. (2010). Effect of nitroethane, dimethyl-2-nitroglutarate and 2-nitro-methyl-propionate on ruminal methane production and hydrogen balance *in vitro. Bioresour*. Technol. 101, 5345–5349. 10.1016/j.biortech.2009.11.108 20194018

[B9] Anderson R. C. Jung Y. S. Genovese K. J. McReynolds J. L. Callaway T. R. Edrington T. S. (2006b). Low level nitrate or nitroethane preconditioning enhances the bactericidal effect of suboptimal experimental chlorate treatment against *Escherichia coli* and *Salmonella* Typhimurium but not *Campylobacter* in swine. Foodborne Pathog. Dis. 3, 461–465. 10.1089/fpd.2006.3.461 17199529

[B10] Anderson R. C. Jung Y. S. Nisbet D. J. (2007). Use of 2-Nitropropanal, 2-Nitroethane, and 2-Nitroethanol for Control of Microbial Pathogens. U.S. Patent No 7, 179, 244 B1. Washington, DC: U.S. Patent and Trademark Office.

[B11] Anderson R. C. Krueger N. A. Stanton T. B. Callaway T. R. Edrington T. S. Harvey R. B. (2008). Effects of select nitrocompounds on *in vitro* ruminal fermentation during conditions of limiting or excess added reductant. Bioresour. Technol. 99, 8655–8661. 10.1016/j.biortech.2008.04.064 18538564

[B12] Anderson R. C. Majak W. Rassmussen M. A. Callaway T. R. Beier R. C. Nisbet D. J.. (2005). Toxicity and metabolism of the conjugates of 3-nitropropanol and 3-nitropropionic acid in forages poisonous to livestock. J. Agric. Food Chem. 53, 2344–2350. 10.1021/jf040392j 15769179

[B13] Anderson R. C. Rasmussen M. A. (1998). Use of a novel nitrotoxin-metabolizing bacterium to reduce ruminal methane production. Bioresour. Technol. 64, 89–95. 10.1016/S0960-8524(97)00184-3

[B14] Anderson R. C. Rasmussen M. A. Allison M. J. (1993). Metabolism of the plant toxins nitropropionic acid and nitropropanol by ruminal microorganisms. Appl. Environ. Microbiol. 59, 3056–3061. 8215375 10.1128/aem.59.9.3056-3061.1993 PMC182406

[B15] Anderson R. C. Rasmussen M. A. Allison M. J. (1996). Enrichment and isolation of a nitropropanol-metabolizing bacterium from the rumen. Appl. Environ. Microbiol. 62, 3885–3886. 8837447 10.1128/aem.62.10.3885-3886.1996 PMC168200

[B16] Anderson R. C. Rasmussen M. A. DiSpirito A. A. Allison M. J. (1997). Characteristics of a nitropropanol-metabolizing bacterium isolated from the rumen. Can. J. Microbiol. 43, 617–624. 10.1139/m97-088 9246740

[B17] Angermaier L. Simon H. (1983). On the reduction of aliphatic and aromatic nitro compounds by *Clostridia*, the role of ferredoxin and its stabilization. Hoppe-Seylers Z. Für Physiol. Chem. 364, 961–975. 10.1515/bchm2.1983.364.2.961 6313511

[B18] Arai H. Mizutani M. Igarashi Y. (2003). Transcriptional regulation of the *nos* genes for nitrous oxide reductase in *Pseudomonas aeruginosa*. Microbiology 149, 29–36. 10.1099/mic.0.25936-0 12576577

[B19] Asanuma N. Iwamoto M. Kawato M. Hino T. (2002). Numbers of nitrate-reducing bacteria in the rumen as estimated by competitive polymerase chain reaction. Anim. Sci. J. 73, 199–205. 10.1046/j.1344-3941.2002.00028.x

[B20] Asanuma N. Yokoyama S. Hino T. (2015). Effects of nitrate addition to a diet on fermentation and microbial populations in the rumen of goats, with special reference to *Selenomonas ruminantium* having the ability to reduce nitrate and nitrite. Anim. Sci. J. 86, 378–384. 10.1111/asj.12307 25439583

[B21] Bozic A. K. Anderson R. C. Carstens G. E. Ricke S. C. Callaway T. R. Yokoyama M. T.. (2009). Effects of the methane-inhibitors nitrate, nitroethane, lauric acid, Lauricidin and the Hawaiian marine algae *Chaetoceros* on ruminal fermentation *in vitro*. Bioresour. Technol. 100, 4017–4025. 10.1016/j.biortech.2008.12.061 19362827

[B22] Brown E. G. Anderson R. C. Carstens G. E. Gutierrez-Banuelos H. McReynolds J. L. Slay L. J. (2011). Effects of oral nitroethane administration on enteric methane emissions and ruminal fermentation in cattle. Anim. Feed Sci. Technol. 166–167, 275–281. 10.1016/j.anifeedsci.2011.04.017

[B23] Brulc J. M. Antonopoulos D. A. Miller M. E. B. Wilson M. K. Yannarell A. C. Dinsdale E. A.. (2009). Gene-centric metagenomics of the fiber-adherent bovine rumen microbiome reveals forage specific glycoside hydrolases. Proc. Natl. Acad. Sci. U.S.A. 106, 1948–1953. 10.1073/pnas.0806191105 19181843 PMC2633212

[B24] Bryant M. P. (1959). Bacterial species of the rumen. Bacteriol. Rev. 23, 125–153. 13805451 10.1128/br.23.3.125-153.1959 PMC181027

[B25] Burrows G. E. Horn G. W. Mcnew R. W. Croy L. I. Keeton R. D. Kyle J. (1987). The prophylactic effect of corn supplementation on experimental nitrate intoxication in cattle. J. Anim. Sci. 64, 1682–1689. 3597182 10.2527/jas1987.6461682x

[B26] Callaghan M. J. Tomkins N. W. Benu I. Parker A. J. (2014). How feasible is it to replace urea with nitrates to mitigate greenhouse gas emissions from extensively managed beef cattle? Anim. Prod. Sci. 54, 1300–1304. 10.1071/an14270

[B27] Cammack R. Joannou C. L. Cui X. Y. Martinez C. T. Maraj S. R. Hughes M. N. (1999). Nitrite and nitrosyl compounds in food preservation. Biochim. Biophys. Acta 1411, 475–488. 10.1016/S0005-2728(99)00033-X 10320676

[B28] Carver L. A. Pfander W. H. (1974). Some metabolic aspects of urea and/or potassium nitrate utilization by sheep. J. Anim. Sci. 38, 410–416. 4812295 10.2527/jas1974.382410x

[B29] Cheng K.-J. Phillippe R. C. Kozub G. C. Majak W. Costerton J. W. (1985). Induction of nitrate and nitrite metabolism in bovine rumen fluid and the transfer of this capacity to untreated animals. Can. J. Anim. Sci. 65, 647–652. 10.4141/cjas85-076

[B30] de Raphélis-Soissan V. Li L. Godwin I. R. Barnett M. C. Perdok H. B. Hegarty R. S. (2014). Use of nitrate and *Propionibacterium acidipropionici* to reduce methane emissions and increase wool growth of Merino sheep. Anim. Prod. Sci. 54, 1860–1866. 10.1071/AN14329

[B31] Dimitrijevic M. Anderson R. C. Callaway T. R. Jung Y. S. Harvey R. B. Ricke S. C. (2006). Inhibitory effect of select nitrocompounds on growth and survivability of *Listeria monocytogenes in vitro*. J. Food Prot. 69, 1061–1065. 16715805 10.4315/0362-028x-69.5.1061

[B32] Duncan S. H. Doherty C. J. Govan J. R. Neogrady S. Galfi P. Stewart C. S. (1999). Characteristics of sheep-rumen isolates of *Pseudomonas aeruginosa* inhibitory to the growth of *Escherichia coli* O157. FEMS Microbiol. Lett. 180, 305–310. 10.1111/j.1574-6968.1999.tb08810.x 10556726

[B33] Ellis J. Hillman K. Williams A. G. Lloyd D. (1990). Hydrogen production by rumen ciliate protozoa, in Microbiology and Biochemistry of Strict Anaerobes Involved in Interspecies Hydrogen Transfer, Federation of European Microbiological Societies Symposium Series, eds Bélaich J.-P. Bruschi M. Garcia J.-L. (New York, NY: Springer), 377–379. 10.1007/978-1-4613-0613-9_38

[B34] El-Zaiat H. M. Araujo R. C. Soltan Y. A. Morsy A. S. Louvandini H. Pires A. V.. (2014). Encapsulated nitrate and cashew nut shell liquid on blood and rumen constituents, methane emission, and growth performance of lambs. J. Anim. Sci. 92, 2214–2224. 10.2527/jas.2013-7084 24663200

[B35] Farra P. A. Satter L. D. (1971). Manipulation of the ruminal fermentation. III. Effect of nitrate on ruminal volatile fatty acid production and milk composition. J. Dairy Sci. 54, 1018–1024. 10.3168/jds.S0022-0302(71)85965-9

[B36] Gutierrez-Bañuelos H. Anderson R. C. Carstens G. E. Slay L. J. Ramlachan N. Horrocks S. M.. (2007). Zoonotic bacterial populations, gut fermentation characteristics and methane production in feedlot steers during oral nitroethane treatment and after the feeding of an experimental chlorate product. Anaerobe 13, 21–31. 10.1016/j.anaerobe.2006.11.002 17208022

[B37] Gutierrez-Bañuelos H. Anderson R. C. Carstens G. E. Tedeschi L. O. Pinchak W. E. Cabrera-Diaz E.. (2008). Effects of nitroethane and monensin on ruminal fluid fermentation characteristics and nitrocompound-metabolizing bacterial populations. J. Agric. Food Chem. 56, 4650–4658. 10.1021/jf800756c 18491914

[B38] Haisan J. Sun Y. Guan L. L. Beauchemin K. A. Iwaasa A. Duval S.. (2014). The effects of feeding 3-nitrooxypropanol on methane emissions and productivity of Holstein cows in mid lactation. J. Dairy Sci. 97, 3110–3119. 10.3168/jds.2013-7834 24630651

[B39] Hasan S. M. Hall J. B. (1975). The physiological function of nitrate reduction in *Clostridium perfringens*. J. Gen. Microbiol. 87, 120–128. 10.1099/00221287-87-1-120 166143

[B40] Hegarty R. S. Gerdes R. (1999). Hydrogen production and transfer in the rumen. Recent Adv. Anim. Nutr. Australia 12, 37–44.

[B41] Hibberd C. A. Rehberger T. G. Swartzlander J. Parrott T. (1994). Utilization of high nitrate forages by beef cows, dairy cows and stocker calves, in Conference Proceedings Management of High Nitrate Forages for Beef and Dairy Cattle. Available online at: http://beefextension.com/proceedings/nitrates93/nitrates93_f.pdf

[B42] Hristov A. N. Oh J. Giallongo F. Frederick T. W. Harper M. T. Weeks H. L.. (2015). An inhibitor persistently decreased enteric methane emission from dairy cows with no negative effect on milk production. Proc. Natl. Acad. Sci. U.S.A. 112, 10663–10668. 10.1073/pnas.1504124112 26229078 PMC4553761

[B43] Hulshof R. B. A. Berndt A. Gerrits W. J. J. Dijkstra J. van Zijderveld S. M. Newbold J. R.. (2012). Dietary nitrate supplementation reduces methane emission in beef cattle fed sugarcane-based diets. J. Anim. Sci. 90, 2317–2323. 10.2527/jas.2011-4209 22287674

[B44] Iwamoto M. Asanuma N. Hino T. (2001). Effects of protozoa on nitrate and nitrite reduction in ruminal microbiota. Kanto J. Anim. Sci. 51, 9–15.

[B45] Iwamoto M. Asanuma N. Hino T. (2002). Ability of *Selenomonas ruminantium, Veillonella parvula*, and *Wolinella succinogenes* to reduce nitrate and nitrite with special reference to the suppression of ruminal methanogenesis. Anaerobe 8, 209–215. 10.1006/anae.2002.0428

[B46] Jainudeen M. R. Hansel W. Davison K. L. (1964). Nitrate toxicity in dairy heifers. 2. Erythropoietic responses to nitrate ingestion during pregnancy. J. Dairy Sci. 47, 1382–1387. 10.3168/jds.S0022-0302(64)88922-0 14277424

[B47] Jamieson N. D. (1959). Rumen nitrate metabolism and the changes occurring in the composition of the rumen volatile fatty acids of grazing sheep. New Zealand J. Agric. Res. 2, 314–328. 10.1080/00288233.1959.10420320

[B48] Johnson K. A. Johnson D. E. (1995). Methane emissions from cattle. J. Anim. Sci. 73, 2483–2492. 8567486 10.2527/1995.7382483x

[B49] Jones G. A. (1972). Dissimilatory metabolism of nitrate by the rumen microbiota. Can. J. Microbiol. 18, 1783–1787. 10.1139/m72-279 4675328

[B50] Jung Y. S. Anderson R. C. Byrd J. A. Edrington T. S. Moore R. W. Callaway T. R.. (2003). Reduction of *Salmonella* Typhimurium in experimentally challenged broilers by nitrate adaptation and chlorate supplementation in drinking water. J. Food Prot. 66, 660–663. 12696692 10.4315/0362-028x-66.4.660

[B51] Jung Y. S. Anderson R. C. Callaway T. R. Edrington T. S. Genovese K. J. Harvey R. B. (2004a). Inhibitory activity of 2-nitropropanol against select food-borne pathogens *in vitro*. Lett. Appl. Microbiol. 39, 471–476. 10.1111/j.1472-765X.2004.01613.x 15482440

[B52] Jung Y. S. Anderson R. C. Edrington T. S. Genovese K. J. Byrd J. A. Callaway T. R.. (2004b). Experimental use of 2-nitropropanol for reduction of *Salmonella* Typhimurium in the ceca of broiler chicks. J. Food Prot. 67, 1945–1947. 15453586 10.4315/0362-028x-67.9.1945

[B53] Kalachniuk H. I. Hrabovens'kyi I. I. Savka O. H. Nezhlukchenko T. I. Shmidt R. M. Venhrin I. D. (1978). Evaluation of Metabolic Activity of Rumen Content. Visnyk Silskohospodarskoi Nauky. Available online at: http://agris.fao.org/agris-search/search.do?recordID=US201302841900 (Accessed November 17, 2015).

[B54] Kaspar H. F. (1982). Nitrite reduction to nitrous oxide by propionibacteria: Detoxication mechanism. Arch. Microbiol. 133, 126–130. 10.1007/BF00413525

[B55] Kaspar H. F. Tiedje J. M. (1981). Dissimilatory reduction of nitrate and nitrite in the bovine rumen - nitrous-oxide production and effect of acetylene. Appl. Environ. Microbiol. 41, 705–709. 7224631 10.1128/aem.41.3.705-709.1981 PMC243764

[B56] Kim W. K. Anderson R. C. Ratliff A. L. Nisbet D. J. Ricke S. C. (2005). Growth inhibition by nitrocompounds of selected uric-acid utilizing microorganisms isolated from poultry manure. J. Environ. Sci. Health. B 41, 97–107. 10.1080/03601230500234950 16393898

[B57] Kim W. K. Weeks L. J. Anderson R. C. Nisbet D. J. Dunkley K. Ricke S. C. (2009). Effects of nitrocompounds on uric acid-utilizing microorganisms, nitrogen retention, and microbial community in laying hen manure. J. Environ. Sci. Health. B 44, 403–406. 10.1080/03601230902801133 19365758

[B58] Lana R. P. Russell J. B. Van Amburgh M. E. (1998). The role of pH in regulating ruminal methane and ammonia production. J. Anim. Sci. 76, 2190–2196. 9734871 10.2527/1998.7682190x

[B59] Lee C. Araujo R. C. Koenig K. M. Beauchemin K. A. (2015a). Effects of encapsulated nitrate on eating behavior, rumen fermentation, and blood profile of beef heifers fed restrictively or *ad libitum*. J. Anim. Sci. 93, 2405–2418. 10.2527/jas.2014-8851 26020336

[B60] Lee C. Araujo R. C. Koenig K. M. Beauchemin K. A. (2015b). Effects of encapsulated nitrate on enteric methane production and nitrogen and energy utilization in beef heifers. J. Anim. Sci. 93, 2391–2404. 10.2527/jas.2014-8845 26020335

[B61] Lee C. Beauchemin K. A. (2014). A review of feeding supplementary nitrate to ruminant animals: nitrate toxicity, methane emissions, and production performance. Can. J. Anim. Sci. 94, 557–570. 10.4141/cjas-2014-069

[B62] Lee S. J. Song H. Lee S. Y. (2006). Genome-based metabolic engineering of *Mannheimia succiniciproducens* for succinic acid production. Appl. Environ. Microbiol. 72, 1939–1948. 10.1128/AEM.72.3.1939-1948.2006 16517641 PMC1393240

[B63] Leng R. A. (2008). The potential of feeding nitrate to reduce enteric methane production in ruminants, A Report to the Departmernt of Climate Change Commonwealth Government of Australia. Canberra, ACT.

[B64] Leng R. A. (2014). Interactions between microbial consortia in biofilms: a paradigm shift in rumen microbial ecology and enteric methane mitigation. Anim. Prod. Sci. 54, 519–543. 10.1071/AN13381

[B65] Leng R. A. Preston T. R. Inthapanya S. (2012). Biochar reduces enteric methane and improves growth and feed conversion in local “Yellow” cattle fed cassava root chips and fresh cassava foliage. Livest. Res. Rural Dev. 24 Available online at: http://www.lrrd.org/lrrd24/11/leng24199.htm

[B66] Lewis D. (1950). The reduction of nitrate by rumen bacteria. J. Gen. Microbiol. 4, 175–180. 14778960

[B67] Lewis D. (1951). The metabolism of nitrate and nitrite in the sheep. 2. Hydrogen donators in nitrate reduction by rumen micro-organisms *in vitro. Biochem*. J. 49, 149–153. 10.1042/bj0490149 PMC1197474 14858300

[B68] Li L. Davis J. Nolan J. Hegarty R. (2012). An initial investigation on rumen fermentation pattern and methane emission of sheep offered diets containing urea or nitrate as the nitrogen source. Anim. Prod. Sci. 52, 653–658. 10.1071/an1125424511695

[B69] Lin M. Guo W. Meng Q. X. Stevenson D. M. Weimer P. J. Schaefer D. M. (2013a). Changes in rumen bacterial community composition in steers in response to dietary nitrate. Appl. Microbiol. Biotechnol. 97, 8719–8727. 10.1007/s00253-013-5143-z 23955503

[B70] Lin M. Schaefer D. M. Guo W. S. Ren L. P. Meng Q. X. (2011). Comparisons of *in vitro* nitrate reduction, methanogenesis, and fermentation acid profile among rumen bacterial, protozoal and fungal fractions. Asian Australas. J. Anim. Sci. 24, 471–478. 10.5713/ajas.2011.10288

[B71] Lin M. Schaefer D. M. Zhao G. Q. Meng Q. X. (2013b). Effects of nitrate adaptation by rumen inocula donors and substrate fiber proportion on *in vitro* nitrate disappearance, methanogenesis, and rumen fermentation acid. Animal 7, 1099–1105. 10.1017/S1751731113000116 23391259

[B72] Looper C. G. Stallcup O. T. Reed F. E. (1959). Deamination of amino acids *in vivo* by rumen microorganisms. J. Anim. Sci. 18, 954–958.

[B73] Lund P. Dahl R. Yang H. J. Hellwing A. L. F. Cao B. B. Weisbjerg M. R. (2014). The acute effect of addition of nitrate on *in vitro* and *in vivo* methane emission in dairy cows. Anim. Prod. Sci. 54, 1432–1435. 10.1071/an14339

[B74] Majak W. (1992). Further enhancement of 3-nitropropanol detoxification by ruminal bacteria in cattle. Can. J. Anim. Sci. 72, 863–870. 10.4141/cjas92-098

[B75] Majak W. Cheng K.-J. (1984). Induction and transfer of the microbial capacity to degrade nitrotoxins in the rumen. Can. J. Anim. Sci. 64, 33–34. 10.4141/cjas84-143

[B76] Marais J. P. Therion J. J. Mackie R. I. Kistner A. Dennison C. (1988). Effect of nitrate and its reduction products on the growth and activity of the rumen microbial-population. Br. J. Nutr. 59, 301–313. 10.1079/BJN19880037 3358930

[B77] Marounek M. Wallace R. J. (1984). Influence of culture *E*_h_ on the growth and metabolism of the rumen bacteria *Selenomonas ruminantium, Bacteroides amylophilus, Bacteroides succinogenes* and *Streptococcus bovis* in batch culture. Microbiology 130, 223–229. 10.1099/00221287-130-2-223

[B78] Martínez-Fernández G. Abecia L. Arco A. Cantalapiedra-Hijar G. Martín-García A. I. Molina-Alcaide E.. (2014). Effects of ethyl-3-nitrooxy propionate and 3-nitrooxypropanol on ruminal fermentation, microbial abundance, and methane emissions in sheep. J. Dairy Sci. 97, 3790–3799. 10.3168/jds.2013-7398 24731636

[B79] Miller T. L. (1995). Ecology of methane production and hydrogen sinks in the rumen, in Ruminant Physiology: Digestion, Metabolism, Growth and Reproduction, eds Engelhardt W. V. Leonhard-Marek S. Breves G. Giesecke D. (Stuttgart: Ferdinand Enke Verlag), 317–331.

[B80] Mitsumori M. Ajisaka N. Tajima K. Kajikawa H. Kurihara M. (2002). Detection of Proteobacteria from the rumen by PCR using methanotroph-specific primers. Lett. Appl. Microbiol. 35, 251–255. 10.1046/j.1472-765X.2002.01172.x 12180951

[B81] Moreno-Vivián C. Cabello P. Martínez-Luque B. R. Blasco R. Castillo F. (1999). Prokaryotic nitrate reduction: molecular properties and functional distinction among bacterial nitrate reductases. J. Bacteriol. 181, 6573–6584. 10542156 10.1128/jb.181.21.6573-6584.1999 PMC94119

[B82] Neumeier C. Wang Q. Castillo A. Zhao Y. Pan Y. Mitloehner F. (2014). Effects of Dietary Nitrate Supplementation on Enteric Methane and Nitrous Oxide Emissions from Beef Cattle. in ASAS. Available online at: https://asas.confex.com/asas/jam2014/webprogram/Paper7717.html (Accessed August 3, 2015).

[B83] Newbold J. R. van Zijderveld S. M. Hulshof R. B. A. Fokkink W. B. Leng R. A. Terencio P.. (2014). The effect of incremental levels of dietary nitrate on methane emissions in Holstein steers and performance in Nelore bulls. J. Anim. Sci. 92, 5032–5040. 10.2527/jas.2014-7677 25349351

[B84] Nishino S. F. Shin K. A. Payne R. B. Spain J. C. (2010). Growth of bacteria on 3-nitropropionic acid as a sole source of carbon, nitrogen, and energy. Appl. Environ. Microbiol. 76, 3590–3598. 10.1128/AEM.00267-10 20382807 PMC2876434

[B85] Nolan J. V. Hegarty R. S. Hegarty J. Godwin I. R. Woodgate R. (2010). Effects of dietary nitrate on fermentation, methane production and digesta kinetics in sheep. Anim. Prod. Sci. 50, 801–806. 10.1071/AN09211

[B86] Pal K. Patra A. K. Sahoo A. Mandal G. P. (2014). Effect of nitrate and fumarate in *Prosopis cineraria* and *Ailanthus excelsa* leaves-based diets on methane production and rumen fermentation. Small Rumin. Res. 121, 168–174. 10.1016/j.smallrumres.2014.08.004

[B87] Patra A. K. Yu Z. T. (2014). Combinations of nitrate, saponin, and sulfate additively reduce methane production by rumen cultures *in vitro* while not adversely affecting feed digestion, fermentation or microbial communities. Bioresour. Technol. 155, 129–135. 10.1016/j.biortech.2013.12.099 24440491

[B88] Petersen S. O. Hellwing A. L. F. Brask M. Højberg O. Poulsen M. Zhu Z.. (2015). Dietary nitrate for methane mitigation leads to nitrous oxide emissions from dairy cows. J. Environ. Qual. 44, 1063–1070. 10.2134/jeq2015.02.0107 26437087

[B89] Pfennig N. Widdel F. Trüper H. G. (1981). The dissimilatory sulfate-reducing bacteria, in The Prokaryotes, eds Starr M. P. Stolp H. Trüper H. G. Balows A. Schlegel H. G. (Berlin; Heidelberg: Springer), 926–940. Available online at: http://link.springer.com/chapter/10.1007/978-3-662-13187-9_74 (Accessed November 17, 2015).

[B90] Phuong L. E. (2012). Mitigation of Methane Production from Ruminants: Effect of Nitrate and Urea on Methane Production in an In vitro System and on Growth Performance and Methane Emissions in Growing Cattle. Master's thesis, Can Tho University.

[B91] Prakash D. (2014). Methyl-coenzyme M Reductase: Elucidating the Process of Activation and Study of the Effect of the Methanogenesis Inhibitor 3-Nitrooxypropanol. PhD Dissertation, Aubrun University.

[B92] Reynolds C. K. Humphries D. J. Kirton P. Kindermann M. Duval S. Steinberg W. (2014). Effects of 3-nitrooxypropanol on methane emission, digestion, and energy and nitrogen balance of lactating dairy cows. J. Dairy Sci. 97, 3777–3789. 10.3168/jds.2013-7397 24704240

[B93] Romero-Pérez A. Okine E. K. Guan L. L. Duval S. M. Kindermann M. Beauchemin K. A. (2015a). Effects of 3-nitrooxypropanol on methane production using the rumen simulation technique (Rusitec). Anim. Feed Sci. Technol. 209, 89–109. 10.1016/j.anifeedsci.2015.09.002 28992012

[B94] Romero-Perez A. Okine E. K. McGinn S. M. Guan L. L. Oba M. Duval S. M.. (2014). The potential of 3-nitrooxypropanol to lower enteric methane emissions from beef cattle. J. Anim. Sci. 92, 4682–4693. 10.2527/jas.2014-7573 25184838

[B95] Romero-Perez A. Okine E. K. McGinn S. M. Guan L. L. Oba M. Duval S. M.. (2015b). Sustained reduction in methane production from long-term addition of 3-nitrooxypropanol to a beef cattle diet. J. Anim. Sci. 93, 1780–1791. 10.2527/jas.2014-8726 26020199

[B96] Russell J. B. (1998). The importance of pH in the regulation of ruminal acetate to propionate ratio and methane production *in vitro*. J. Dairy Sci. 81, 3222–3230. 10.3168/jds.S0022-0302(98)75886-2 9891267

[B97] Russell J. B. (2002). Rumen Microbiology and its Role in Ruminant Nutrition. Ithaca, NY: Cornell University.

[B98] Russell J. B. Rychlik J. L. (2001). Factors that alter rumen microbial ecology. Science 292, 1119–1122. 10.1126/science.1058830 11352069

[B99] Sar C. Mwenya B. Pen B. Takaura K. Morikawa R. Tsujimoto A.. (2005). Effect of ruminal administration of *Escherichia coli* wild type or a genetically modified strain with enhanced high nitrite reductase activity on methane emission and nitrate toxicity in nitrate-infused sheep. Br. J. Nutr. 94, 691–697. 10.1079/BJN20051517 16277770

[B100] Sar C. Mwenya B. Pen B. Takaura K. Morikawa R. Tsujimoto A. (2006). Effect of wild type *Escherichia coli* W3110 or *Escherichia coli nir*-Ptac on methane emission and nitrate toxicity in nitrate-treated sheep. Int. Congr. Ser. 1293, 193–196. 10.1016/j.ics.2006.03.015

[B101] Sar C. Santoso B. Gamo Y. Kobayashi T. Shiozaki S. Kimura K. (2004). Effects of combination of nitrate with beta 1-4 galacto-oligosaccharides and yeast (*Candida kefyr*) on methane emission from sheep. Asian Australas. J. Anim. Sci. 17, 73–79. 10.5713/ajas.2004.73

[B102] Shi C. X. Meng Q. X. Hou X. Z. Ren L. P. Zhou Z. M. (2012). Response of ruminal fermentation, methane production and dry matter digestibility to microbial source and nitrate addition level in an *in vitro* incubation with rumen microbes obtained from wethers. J. Anim. Vet. Adv. 11, 3334–3341. 10.3923/javaa.2012.3334.3341

[B103] Silivong P. Preston T. R. Van Man N. (2012). Effect of supplements of potassium nitrate or urea as sources of NPN on methane production in an *in vitro* system using molasses and Paper mulberry or Muntingia foliages as the substrate. Livest. Res. Rural Dev. 17, 4–12.

[B104] Sophea I. V. Preston T. R. (2011). Effect of different levels of supplementary potassium nitrate replacing urea on growth rates and methane production in goats fed rice straw, mimosa foliage and water spinach. Livest. Res. Rural Dev. 23.

[B105] Stewart V. (1988). Nitrate respiration in relation to facultative metabolism in enterobacteria. Microbiol. Rev. 52, 190–232. 3045516 10.1128/mr.52.2.190-232.1988 PMC373136

[B106] Sutherland T. M. (1977). The control and manipulation of rumen fermentation. Rec. Adv. Anim. Nutr. Aust. 1, 110–129.

[B107] Takahashi J. Ikeda M. Matsuoka S. Fujita H. (1998). Prophylactic effect of L-cysteine to acute and subclinical nitrate toxicity in sheep. Anim. Feed Sci. Technol. 74, 273–280. 10.1016/S0377-8401(98)00176-X

[B108] Takahashi J. Masuko T. Endo S. Dodo K. Fujita H. (1980). Effects of dietary protein and energy levels on the reduction of nitrate and nitrite in the rumen and methemoglobin formation in sheep. Anim. Sci. J. 51, 626–631. 10.2508/chikusan.51.626

[B109] Takahashi J. Young B. (1991). Prophylactic effect of L-cysteine on nitrate-induced alterations in respiratory exchange and metabolic-rate in sheep. Anim. Feed Sci. Technol. 35, 105–113. 10.1016/0377-8401(91)90103-Y

[B110] Thanh V. D. Thu N. V. Preston T. R. (2012). Effect of potassium nitrate or urea as NPN source and levels of Mangosteen peel on *in vitro* gas and methane production using molasses, *Operculina turpethum* and *Brachiaria mutica* as substrate. Livest. Res. Rural Dev. 24 Available online at: http://www.lrrd.org/lrrd24/4/thanh24063.htm

[B111] Thauer R. K. Jungermann K. Decker K. (1977). Energy conservation in chemotrophic anaerobic bacteria. Bacteriol. Rev. 41, 100–180. 860983 10.1128/br.41.1.100-180.1977 PMC413997

[B112] Tillman A. D. Sheriha G. M. Sirny R. J. (1965). Nitrate reduction studies with sheep. J. Anim. Sci. 24, 1140–1146.

[B113] Ungerfeld E. M. Kohn R. A. (2006). The role of thermodynamics in the control of ruminal fermentation, in Ruminant Physiology, eds Sejrsen K. Hvelplund T. Nielsen M. O. (Wageningen, DC: Wageningen Academic Publishers), 55–85.

[B114] US EPA (2012). U.S. Greenhouse Gas Inventory Report. Available online at: http://www.epa.gov/climatechange/ghgemissions/usinventoryreport.html (Accessed July 20, 2015).

[B115] Van Kessel J. A. S. Russell J. B. (1996). The effect of pH on ruminal methanogenesis. FEMS Microbiol. Ecol. 205–210. 10.1111/j.1574-6941.1996.tb00319.x26779340

[B116] Van Nevel C. J. Demeyer D. I. (1996). Control of rumen methanogenesis. Environ. Monit. Assess 42, 73–97. 10.1007/BF00394043 24193494

[B117] van Zijderveld S. M. Gerrits W. J. J. Apajalahti J. A. Newbold J. R. Dijkstra J. Leng R. A.. (2010). Nitrate and sulfate: Effective alternative hydrogen sinks for mitigation of ruminal methane production in sheep. J. Dairy Sci. 93, 5856–5866. 10.3168/jds.2010-3281 21094759

[B118] van Zijderveld S. M. Gerrits W. J. J. Dijkstra J. Newbold J. R. Hulshof R. B. A. Perdok H. B. (2011). Persistency of methane mitigation by dietary nitrate supplementation in dairy cows. J. Dairy Sci. 94, 4028–4038. 10.3168/jds.2011-4236 21787938

[B119] Velazco J. I. Cottle D. J. Hegarty R. S. (2014). Methane emissions and feeding behaviour of feedlot cattle supplemented with nitrate or urea. Anim. Prod. Sci. 54, 1737–1740. 10.1071/AN1434526922524

[B120] Yoshida J. Nakamura Y. Nakamura R. (1982). Effects of protozoal fraction and lactate on nitrate metabolism of microorganisms in sheep rumen. Jpn. J. Zootech. Sci. 53, 677–685. 10.2508/chikusan.53.677

[B121] Zhang Y. Long R. J. Warzecha C. M. Coverdale J. A. Latham E. A. Hume M. E.. (2014). Characterization of bovine ruminal and equine cecal microbial populations enriched for enhanced nitro-toxin metabolizing activity. Anaerobe 26, 7–13. 10.1016/j.anaerobe.2013.12.001 24374155

[B122] Zhao L. Meng Q. Ren L. Liu W. Zhang X. Huo Y.. (2015). Effects of nitrate addition on rumen fermentation, bacterial biodiversity and abundance. Asian-Australas. J. Anim. Sci. 28, 1433–1441. 10.5713/ajas.15.0091 26194220 PMC4554850

[B123] Zhou Z. Meng Q. Yu Z. (2011). Effects of methanogenic inhibitors on methane production and abundances of methanogens and cellulolytic bacteria in *in vitro* ruminal cultures. Appl. Environ. Microbiol. 77, 2634–2639. 10.1128/AEM.02779-10 21357427 PMC3126374

[B124] Zhou Z. Yu Z. Meng Q. (2012). Effects of nitrate on methane production, fermentation, and microbial populations in *in vitro* ruminal cultures. Bioresour. Technol. 103, 173–179. 10.1016/j.biortech.2011.10.013 22047657

[B125] Zumft W. G. Kroneck P. M. H. (2007). Respiratory transformation of nitrous oxide(N_2_O) to dinitrogen by bacteria and archaea. Adv. Microb. Physiol. 52, 107–227. 10.1016/S0065-2911(06)52003-X 17027372

